# Red blood cell transfusion practices in extracorporeal membrane oxygenation: A single‐center study

**DOI:** 10.1111/tme.13154

**Published:** 2025-07-04

**Authors:** Shailesh Balasubramanian, Mahmoud Alwakeel, Divyajot Sadana, Mani Latifi, Chase Donaldson, Brett Wakefield, Edward Soltesz, Kenneth McCurry, Sudhir Krishnan

**Affiliations:** ^1^ The Division of Pulmonary and Critical Care Medicine, Clinical Immunology, and Allergy David Geffen School of Medicine at UCLA Los Angeles California USA; ^2^ Department of Medicine Duke University Medical Center Durham North Carolina USA; ^3^ Department of Pulmonary and Critical Care Medicine Respiratory Institute, Cleveland Clinic Cleveland Ohio USA; ^4^ Department of Intensive Care and Resuscitation Cleveland Clinic Foundation Cleveland Ohio USA; ^5^ Department of Thoracic and Cardiovascular Surgery Cleveland Clinic Hospital Cleveland Ohio USA

**Keywords:** blood transfusion, ECMO, length of stay, mortality

## Abstract

**Objectives:**

Evaluating blood transfusion practices and their impact on morbidity and mortality across extracorporeal membrane oxygenation (ECMO) configurations.

**Background:**

As ECMO becomes increasingly utilised in critical care, the ideal Hgb level remains uncertain. While guidelines recommend higher levels, emerging evidence suggests potential harm. Our study addresses this gap by investigating the optimal Hgb level for ECMO.

**Methods:**

A retrospective cohort study included all adult patients receiving ECMO between January 2016 and December 2018. The primary outcome assessed the optimal Hgb level associated with reduced ECMO duration and in‐hospital mortality. Multivariable and Cox‐proportional regression analyses were performed.

**Results:**

A total of 306 patients underwent ECMO, with 31 patients having mean Hgb levels 7–7.9 g/dL, 176 patients 8–8.9 g/dL, 72 patients 9–9.9 g/dL, and 27 patients ≥10 g/dL. The mean (SD) age was 56 years (15), with 60.8% male (186/306). ECMO configurations were primarily Venoarterial (VA) (59.8%), followed by Venovenous (VV) (36.9%) and Hybrid (3.3%). The 7–7.9 g/dL Hgb group was associated with longer ECMO duration (mean 17.5 days, coefficient 2.2, 95% CI 0.02–4.4, *p* = 0.048) compared to the ≥10 g/dL group, with no significant mortality differences across Hgb levels. VA ECMO patients had a significantly higher mortality risk than VV ECMO patients (aHR 2.33, 95% CI 1.50–3.60, *p* < 0.001). Blood product use, including RBC and Cryo, was associated with longer ECMO duration, while FFP reduced both duration (coefficient − 0.84, 95% CI ‐1.11–‐0.57, *p* < 0.001) and mortality risk (aHR 0.895, 95% CI 0.818–0.973, *p* = 0.012).

**Conclusion:**

Targeting Hgb level >8 g/dL in ECMO patients may help reduce ECMO duration.

## 1 INTRODUCTION

Extracorporeal Membrane Oxygenation (ECMO) use has increased dramatically over the last decade.^1^ Despite major advances that have improved the safety and ease of its use, ECMO remains a complex, invasive, high‐risk, resource‐intensive and costly life‐saving technology.^2^ ECMO support is associated with high blood utilisation, frequently in the setting of bleeding and coagulopathy, with transfusion requirements reaching upwards of 6 units of packed red blood cells (RBC) per day.^3^, ^4^ RBC transfusion is frequently utilised to supplement oxygen carrying capacity in an attempt to improve oxygen delivery to tissues in refractory hypoxemic conditions.^5^ Current Extracorporeal Life Support (ELSO) guidelines suggest transfusing to target a “normal” haemoglobin (Hgb) level in order to mitigate the risks of high ECMO blood flow rates.^6^ While the physiologic argument for the augmentation of oxygen delivery to tissues cannot be repudiated, any potential benefits from blood transfusions must be weighed against the established risks.

Mazzeffi et al.^7^ showed a 3% increase in mortality per unit of RBC transfused in adult ECMO patients. Multiple other studies have also demonstrated an association between RBC transfusions and worsened outcomes, raising concerns over appropriate transfusion protocols for ECMO patients.^7^, ^8^, ^9^, ^10^, ^11^, ^12^, ^13^ Due to the lack of standardisation, blood transfusion practices vary widely based on tailored institutional practices and indication for ECMO.^3^, ^12^, ^13^, ^14^, ^15^ While several studies support restrictive transfusion strategies (Hgb threshold <7 g/dL) in critically ill patients, there are no randomised controlled studies addressing optimal haemoglobin levels or transfusion practices in ECMO patients.^16^ Furthermore, small sample size and focus on a single ECMO configuration or specific ECMO indications limit the generalisability and applicability of these studies. Also, 33% of transfusion events in the critically ill medical population occur in direct opposition to clinical guidelines with multiple crossovers between threshold of transfusion for the same individual.^17^

We present a real‐world observational study examining blood transfusion practices at a large‐volume tertiary referral ECMO center. We report our transfusion practices for blood transfusion and investigate its impact on morbidity and mortality in the different ECMO configurations. Also, we are exploring the ideal average Hgb level that strikes a balance between minimising over‐transfusion and, simultaneously, achieving improved outcomes characterised by reduced ECMO duration and mortality.

## 2 MATERIALS AND METHODS

### 2.1 Patient selection and study design

This study was approved by the Institutional Review Board at the Cleveland Clinic (IRB # 18‐1339) and conducted in accordance with accepted ethical standards. We conducted a retrospective cohort study using the Cleveland Clinic ECMO database that included all adult patients greater than 18 years old who were hospitalised and received ECMO support between January 2016 and December 2018.

### 2.2 Database information

For all patients we recorded data pertaining to demographics (e.g., age, gender, body mass index (BMI)), Simplified Acute Physiology Score II (SAPS II) at intensive care unit (ICU) admission, and co‐morbidities (e.g., diabetes mellitus (DM), smoking history, hypertension (HTN), atrial fibrillation (A.fib), coronary artery disease (CAD), and venous thromboembolism (VTE)). All relevant haemoglobin (Hgb) and transfusion data, including volume of transfusion, type of transfusion (RBC, platelets (PLT), cryoprecipitate (Cryo), fresh frozen plasma (FFP)), time of transfusion, and pre‐ and post‐transfusion Hb values were extracted using a validated computer algorithm from the institutional electronic medical record system (EMR).^18^ All transfusions were transformed into the number of units using the average millilitres (ml) to unit equivalents for each blood product as follows: 1 RBC = 300 mL, 1 FFP = 250 mL, 1 PLT = 300 mL, and 1 Cryo = 15 mL. Mean Hgb during ECMO was calculated by averaging all whole blood Hgb values in the EMR during the ECMO timeframe. ECMO indication, bleeding, hemolysis, and coagulopathy event data were manually abstracted by a trained registry research abstraction team based on documentation in the electronic health record (EHR). Events were considered positive when the treating team explicitly indicated they were the reason for transfusion.

### 2.3 ECMO characteristics

All patients reviewed in this study received either VV, VA, or a hybrid ECMO configuration (VAV or VVA). Patients whose initial ECMO configuration changed but support was uninterrupted were categorised as hybrid configuration for the purposes of data analysis. The extracorporeal circuits used at our institution include the Maquet Rotaflow, Maquet CardioHelp Rotaflow system, Medtronic Bio‐Console, and Thoratec CentriMag system. The oxygenator circuits used include the Maquet Quadrox and Medtronic MC3 Nautilus. Cannulation is performed using varying cannula sizes from Medtronic, Edwards, Sorin, and Surge.

### 2.4 Data analysis and clinical outcomes

The primary clinical outcome is to assess the optimal average haemoglobin level associated with reduced ECMO duration and in‐hospital mortality. Other outcomes included hospital length of stay (LOS), intensive care unit (ICU) LOS, and mechanical ventilation (MV) duration.

The study cohort was stratified based on the mean Hgb levels during ECMO into four groups: 7–7.9 g/dL, 8–8.9 g/dL, 9–9.9 g/dL, and > 10 g/dL. Patient data were summarised using means and standard deviations (SD) or medians and interquartile ranges (IQR), as appropriate for the distribution of continuous variables. Differences among the groups were analysed using one‐way analysis of variance (ANOVA) or the Kruskal‐Wallis test, based on data normality. For categorical variables, data were summarised using counts and percentages, and differences among the groups were assessed using either Fisher's Exact test or the Chi‐square test.

We conducted a multivariable linear regression analysis to examine the relationship between varying mean Hgb levels throughout ECMO sessions and the duration of ECMO. The Cox proportional hazard model was used to identify the associated adjusted hazard ratio (HR) with different average Hgb levels during ECMO and in‐hospital mortality. In our variable selection process, we employed a stepwise approach with the objective of minimising the Bayesian Information Criterion (BIC) and Akaike Information Criterion (AIC), thus helping to prevent the development of an overfitted or biased model.^19^ We excluded patients in hybrid ECMO configuration or who had hemolysis or coagulopathy documented as the reason for transfusion due to their low numbers, as well as those who withdrew from care to avoid bias. We also assessed regression assumptions, including normality, linearity, homoscedasticity, and absence of multicollinearity. ECMO indications were excluded from the model build due to the potential for multi‐classification and strong collinearity with ECMO configuration, which was already included as a more consistently documented covariate. Table S1 provides a detailed summary of the stepwise selection process, highlighting variables retained in the final ECMO duration model (ECMO configuration, haemoglobin group, transfusion volumes [RBC, FFP, cryoprecipitate], mechanical ventilation duration, and SAPS II score) and those excluded due to minimal incremental contribution (e.g., bleeding, comorbidities, and demographics). The results are presented as coefficients for the ECMO duration and adjusted HR for in‐hospital mortality, along with their corresponding 95% confidence intervals (CI). In addition, we calculated the adjusted R‐squared (*R* ^2^) value to provide a measure of the proportion of variability in the dependent variable accounted for by our regression model, while adjusting for the number of predictors. All statistics were performed using the JMP Pro®, Version 17. SAS Institute Inc., Cary, NC, 1989–2023. The level of statistical significance was set at *p* < 0.05 (two‐tailed).

## 3 RESULTS

In the study period, a total of 306 patients underwent ECMO, with 31 patients having mean Hgb levels during the ECMO duration between 7 and 7.9 g/dL, 176 patients between 8 and 8.9 g/dL, 72 patients between 9 and 9.9 g/dL, and 27 patients with levels equal or exceeding 10 g/dL. The mean (SD) age of patients across the groups ranged from 53 to 57 years (*p* = 0.579). The percentage of male patients varied from 48.4% to 64.8% across the groups (*p* = 0.302). Among the groups, VA ECMO was the predominant type across all categories, followed by VV ECMO, with Hybrid ECMO being rare across all groups (Table 1). The duration of ECMO was markedly longer in the 7–7.9 g/dL group, with a mean of 17.5 days, compared to an average of 6–9 days in the other groups (*p* = 0.003). The incidence of bleeding events during ECMO was 51.6% in the 7–7.9 g/dL group, 43.8% in the 8–8.9 g/dL group, 38.9% in the 9–9.9 g/dL group, and 37.0% in the ≥10 g/dL group (*p* = 0.601). High in‐hospital mortality rates were observed consistently across all groups, with a prevalence of 60% (*p* = 0.926). Table 1 summarises the demographics, clinical characteristics, and outcomes.

**TABLE 1:** Baseline characteristics of ECMO patients.

Clinical characteristic	7–7.9 g/dL (*N* = 31)	8–8.9 g/dL (*N* = 176)	9–9.9 g/dL (*N* = 72)	≥ 10 g/dl (*N* = 27)	*p*‐value
Age (mean (SD))	52.77 (14.76)	56.74 (15.34)	55.60 (15.01)	55.00 (15.12)	0.579
Male (%)	15 (48.4)	114 (64.8)	41 (56.9)	16 (59.3)	0.302
BMI (mean (SD))	31.49 (7.97)	30.82 (7.67)	31.76 (8.05)	31.91 (8.14)	0.787
Admission SAPS II (mean (SD))	43.23 (17.61)	40.06 (14.92)	41.43 (18.45)	45.48 (18.15)	0.366
MV assist (%)	3 (9.7)	41 (23.3)	8 (11.1)	4 (14.8)	0.066
MV duration (mean (SD))	33.48 (41.67)	26.54 (38.98)	28.28 (45.64)	13.44 (8.23)	0.253
ECMO configuration (%)					
VA	15 (48.4)	111 (63.1)	43 (59.7)	14 (51.9)	0.366
VV	16 (51.6)	62 (35.2)	24 (33.3)	11 (40.7)	0.301
Hybrid	0 (0.0)	3 (1.7)	5 (6.9)	2 (7.4)	0.074
Comorbidities					
LVEF (mean (SD))	52.65 (12.65)	52.19 (14.92)	51.62 (15.82)	43.63 (19.69)	0.058
Afib (%)	10 (32.3)	55 (31.2)	17 (23.6)	4 (14.8)	0.24
CAD (%)	12 (38.7)	80 (45.5)	28 (38.9)	8 (29.6)	0.394
DM (%)	10 (32.3)	50 (28.4)	15 (20.8)	5 (18.5)	0.402
COPD (%)	7 (22.6)	30 (17.0)	11 (15.3)	8 (29.6)	0.344
HTN (%)	18 (58.1)	94 (53.4)	36 (50.0)	11 (40.7)	0.555
Smoking history (%)	15 (48.4)	84 (47.7)	43 (59.7)	11 (40.7)	0.257
VTE (%)	6 (19.4)	26 (14.8)	9 (12.5)	5 (18.5)	0.782
ECMO indication^a^					0.534
Cardiac failure	15 (48.4%)	78 (44.3%)	35 (48.6%)	13 (48.1%)	
Post‐operative/procedure‐related	7 (22.6%)	67 (38.1%)	19 (26.4%)	6 (22.2%)	
Respiratory failure	6 (19.4%)	21 (11.9%)	12 (16.7%)	5 (18.5%)	
Sepsis/Mixed	3 (9.7%)	10 (5.7%)	6 (8.3%)	3 (11.1%)	
Bleeding event during ECMO (%)^b^	16 (51.6)	77 (43.8)	28 (38.9)	10 (37.0)	0.601
Hemolysis events during ECMO (%)^b^	3 (9.7)	6 (3.4)	4 (5.6)	1 (3.7)	0.831
Coagulopathy events during ECMO (%)^b^	7 (22.6)	25 (14.2)	8 (11.1)	2 (7.4)	0.539
Volume of transfused blood product (mL)					0.058
Cryoprecipitate (median (IQR))	520.0 (275.0–917.0)	550.0 (300.0–858.0)	441.0 (286.0–1096.5)	206.5 (197.5–303.2)	
FFP (median (IQR))	867.5 (458.0–1755.0)	998.0 (567.0–1719.0)	1182.0 (460.0–2103.5)	867.0 (538.8–1077.5)	
PRBC (median (IQR))	4100.0 (1750.0–8709.0)	3836.5 (2100.0–6585.8)	2100.0 (1190.0–4941.5)	2068.5 (1414.2–2914.0)	
Platelet (median (IQR))	1176.0 (537.0–3259.0)	790.0 (488.8–1534.0)	837.0 (405.0–1424.0)	555.0 (252.2–1044.8)	
Outcomes					
ECMO duration (mean (SD))	17.52 (19.82)	8.94 (11.90)	9.28 (12.49)	6.30 (3.11)	0.003
Hospital LOS, days (mean (SD))	43.19 (42.97)	38.84 (50.01)	41.92 (51.30)	22.96 (24.99)	0.323
ICU LOS, days (mean (SD))	35.61 (36.77)	29.24 (41.33)	35.54 (49.61)	15.44 (10.25)	0.156
Withdrawal care	10 (32.3)	51 (29.0)	18 (25.0)	9 (33.3)	0.811
In‐hospital mortality (%)	19 (61.3)	103 (58.5)	45 (62.5)	17 (63.0)	0.926

Abbreviations: A.fib, atrial fibrillation; ARDS, acute respiratory distress syndrome; BMI, body mass index; CAD, coronary artery disease; COPD, chronic obstructive pulmonary disease; DM, diabetes mellitus; ECMO, extracorporeal membrane oxygenation; FFP, fresh frozen plasma; HTN, hypertension; IQR, interquartile range; LOS, length of hospital stay; LVEF, left ventricular ejection fraction; MV, mechanical ventilation; NICM, non‐ischemic cardiomyopathy; PE, pulmonary embolism; PRBC, packed red blood cells; RV, right ventricular; SAPS, simplified acute physiology score; SD, standard deviation; VA, venoarterial; VF/VT, ventricular fibrillation/ventricular tachycardia; VTE, venous thromboembolism; VV, venovenous.

^a^ Patients with multiple indications were classified as ‘Mixed’ if no dominant indication accounted for >80% of their ECMO duration or clinical trajectory, based on chart review.

^b^ Bleeding, hemolysis, and coagulopathy events were considered present if the clinical team explicitly documented them as the reason for transfusion.

Table 2 summarises the multivariable linear regression analysis results investigating the association between different average Hgb levels during ECMO and the duration of ECMO while controlling for other covariates. Average Hgb group of 7–7.9 g/dL was associated with longer duration than ≥10 g/dL group; coefficient 2.2 (95% CI 0.02–4.4, *p* = 0.048). Other average Hgb groups were not statistically significant compared to the ≥10 g/dL group; the coefficient for the 8–8.9 g/dL group was 0.02 (95% CI −0.9 to 0.6, *p* = 0.234), and for the 9–9.9 g/dL group, it was −0.9 (95% CI −2.5 to 0.6, *p* = 0.234). VA configuration was associated with less duration compared to VV ECMO; coefficient − 1.2 (95% CI −2 to –0.3, *p* = 0.009). Both admission SAPS II score (coefficient: 0.1, 95% CI: 0.01–0.11, *p* = 0.012) and duration of mechanical ventilation (coefficient: 0.1, 95% CI: 0.1 to 0.2, *p* < 0.001) were associated with longer ECMO duration. Blood products associated with longer ECMO duration included RBCs (coefficient: 0.38, 95% CI: 0.28 to 0.47, *p* < 0.001) and Cryo (coefficient: 0.05, 95% CI: 0.03 to 0.06, *p* < 0.001), while FFP (coefficient: −0.84, 95% CI: −1.11 to −0.57, *p* < 0.001) was associated with lower ECMO duration. *R* ^2^ for the model was 64.2%.

**TABLE 2:** Multivariable linear regression analysis: Association between average Hgb level during ECMO and the duration of ECMO.

Term	Estimate coefficient	Lower 95%	Upper 95%	*p*‐value
Intercept	−0.974	−3.579	1.631	0.462
ECMO configuration (VA vs. VV)	−1.149	−2.009	−0.290	0.009
Admission SAPSII Score	0.062	0.014	0.110	0.012
Duration of mechanical ventilation (days)	0.144	0.101	0.188	<0.001
No. of RBCs units	0.376	0.285	0.467	<0.001
No. of FFP units	−0.838	−1.106	−0.571	<0.001
No. of cryo units	0.046	0.030	0.062	<0.001
No. of platelets units	0.172	−0.138	0.482	0.274
Hgb group (7–7.9 vs. ≥10)	2.203	0.018	4.387	0.048
Hgb group (8–8.9 vs. ≥10)	−1.252	−2.527	0.022	0.054
Hgb group (9–9.9 vs. ≥10)	−0.940	−2.491	0.612	0.234

Abbreviations: Cryo, cryoprecipitate; ECMO, extracorporeal membrane oxygenation; FFP, fresh frozen plasma; Hgb, haemoglobin; RBC, red blood cell; SAPS, simplified acute physiology score; VA, venoarterial; VV, venovenous.

**FIGURE 1:**
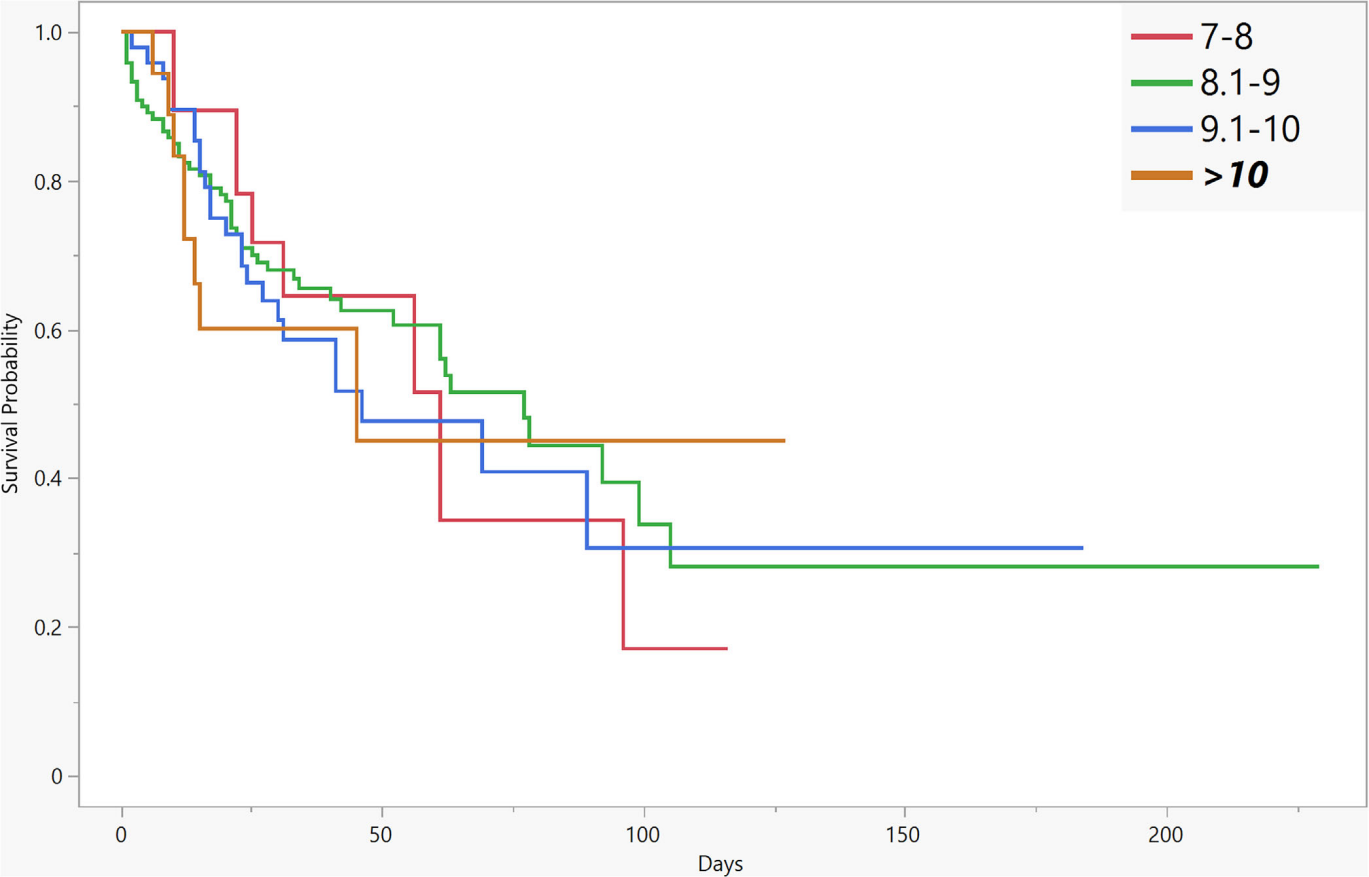
Kaplan–Meier survival analysis: survival probability over time. Survival curves for ECMO patients stratified by mean haemoglobin (Hgb) levels: 7–7.9 g/dL, 8.1–9 g/dL, 9.1–10 g/dL, and > 10 g/dL. The x‐axis represents the number of days on ECMO, and the y‐axis shows survival probability. Statistical analysis using Log‐Rank (χ^2^ = 0.8756, df = 3, *p* = 0.8313) and Wilcoxon (χ^2^ = 1.4264, df = 3, *p* = 0.6993) tests indicated no significant differences in survival between the Hgb groups.

Cox proportional hazard model summarised at Table 3. Different average Hgb levels during ECMO were not associated with an increased risk of mortality, similar to the number of blood products given, except for FFP, which was associated with a 10.5% lower risk of mortality for each given unit (aHR: 0.895, 95% CI: 0.818 to 0.973, *p* = 0.012). The VA ECMO configuration, with a aHR of 2.33 (95% CI: 1.50 to 3.60, *p* < 0.001), was significantly associated with higher mortality risk compared to VV configuration. Additionally, longer duration of mechanical ventilation was associated with decreased mortality risk, with aHR of 0.95 (95% CI: 0.94 to 0.96, *p* < 0.001). Kaplan–Meier survival analysis results, including Log‐Rank (χ^2^ = 0.8756, df = 3, *p* = 0.8313) and Wilcoxon (χ^2^ = 1.4264, df = 3, *p* = 0.6993) tests, are presented in Figure 1.

**TABLE 3:** Cox proportional hazard model assessing the association between different average Hgb levels during ECMO and In‐hospital mortality while controlling for other clinical covariates.

Term	Estimate aHR	Lower 95%	Upper 95%	*p*‐value
ECMO configuration (VA vs. VV)	2.328	1.504	3.603	0.001
Duration of mechanical ventilation (days)	0.952	0.939	0.965	<0.001
No. of RBCs units	1.032	0.999	1.064	0.05
No. of FFP units	0.895	0.818	0.973	0.012
No. of cryo units	1.000	0.996	1.003	0.902
No. of platelets	0.903	0.810	0.999	0.058
Hgb group (7–7.9 vs. ≥10)	0.853	0.326	2.233	0.746
Hgb group (8–8.9 vs. ≥10)	0.843	0.406	1.748	0.646
Hgb group (9–9.9 vs. ≥10)	0.577	0.251	1.323	0.194

Abbreviations: aHR, adjusted hazard ratio; Cryo, cryoprecipitate; ECMO, extracorporeal membrane oxygenation; FFP, fresh frozen plasma; Hgb, haemoglobin; RBC, red blood cell; SAPS, simplified acute physiology score; VA, venoarterial; VV, venovenous.

## 4 DISCUSSION

In our high‐volume tertiary referral ECMO center, adhering to a restrictive average Hgb level of 7–7.9 g/dL, commonly practiced in critical care settings, was associated with prolonged ECMO duration compared to more liberal average haemoglobin levels of greater than 8 g/dL. There was no difference in hospital mortality among all average haemoglobin levels. Furthermore, an increase in the number of RBC transfusions correlated with extended ECMO duration. Although the number of RBC transfusions did not reach statistical significance, a notable trend suggested a potential increase in mortality. Our study is pioneering in the literature by elucidating that targeting an Hgb range of 8–8.9 g/dL in ECMO patients is optimal for averting unnecessary blood transfusions.

The ELSO guidelines continue to advocate for ECMO providers to target elevated Hgb and haematocrit (Hct) levels, specifically aiming for 15 g/dL and >40%, respectively.^20^, ^21^ These recommendations are rooted in the physiological rationale that higher haemoglobin levels enhance oxygen delivery capacity.^22^ While the medical community widely acknowledges the effectiveness of a restrictive blood transfusion protocol (Hgb <7 gm/dL) in critical care settings,^16^ a distinct perspective emerges for ECMO patients.^20^, ^21^ Maintaining higher Hgb levels could potentially help achieve the lowest feasible blood flow, thus mitigating adverse effects like hemolysis and circuit complications, ultimately leading to improved outcomes such as decreased ECMO duration and increased survival rates. Several studies have sought to assess these expert recommendations by investigating various factors, including different blood transfusion thresholds, different haemoglobin levels and the quantity of packed red blood cells transfused, and their impact on mortality rates. Our approach of examining the average maintained Hgb level during ECMO sessions diverged from most of the literature reports due to the inevitable crossover between different transfusion thresholds or protocols, influenced by practitioner variation or physiological exigencies. This makes it challenging to categorise patients into strict threshold cohorts throughout the ECMO duration. The only reliable method to classify patients is through randomised controlled trials or practical protocol application, but even then, compliance remains questionable, and any deviation may introduce bias into the findings. Therefore, we opted for a practical standpoint: irrespective of transfusion reasons or thresholds, does maintaining an average Hgb level at a certain interval affect ECMO duration and mortality rates?

A recent large multicenter prospective observational study, PROTECMO,^23^ stratified patients who received VV ECMO by different average daily Hgb levels into <7 g/dL, 7–8 g/dL, 8–10 g/dL, and >10 g/dL. The study revealed that, in non‐adjusted analysis, only the cohort with less than 7 g/dL Hgb was associated with an increased risk of 28‐day mortality (Odds Ratio (OR) 3.15, 95% CI 2.01–4.93), although this risk was not significant after adjusting for other clinical confounders which align with our findings. The study also observed, in the non‐adjusted analysis, that patients who did not receive blood transfusions had significantly shorter ECMO duration, a finding similarly reported by Grazioli et al.^24^ The study did not explore factors influencing ECMO duration, nor did it investigate the impact of blood transfusion quantity on mortality, focusing solely on VV‐ECMO. Ng et al.^25^ investigated a “restrictive” transfusion threshold (≤8.5 g/dL) versus “liberal” transfusion strategy (>8.5 g/dL) and found no survival benefit. Interestingly, the median Hgb level was 8.3 versus 9.9 in the restrictive versus liberal transfusion groups, mirroring our findings within similar haemoglobin intervals. Shudo et al.^26^ from Stanford University did not identify a survival benefit for Hgb threshold transfusion above or below 10 g/dL. Similarly, Hunsicker et al.^27^ did not find a survival benefit between keeping Hgb above 8 or 10 g/dL among VV ECMO patients for acute respiratory distress syndrome (ARDS). A small study by Voelker et al.^28^ comprising 18 patients on VV‐ECMO for ARDS found no survival benefit between <7 or physiological transfusion triggers. In addition to the small sample size, both groups maintained an average Hgb level between 8 and 9 g/dL, consistent with our findings. Furthermore, consistent with our findings, non‐survivors received a higher number of blood transfusions compared to survivors, with averages of 1.97 ± 1.47 units and 0.96 ± 0.76 units, respectively. While the number of transfused RBCs did not reach statistical significance regarding increased mortality in our study, the trend towards association with mortality resembled findings reported by Martucci et al.^29^ The adjusted odds ratio (OR) for mortality in their study showed a similar trend, with each 100 mL/day increase in RBC transfusion associated with an OR of 1.9 (95% CI: 1.1–3.2). Comparable increases in mortality with higher blood transfusion rates have been reported in both adult and paediatric literature.^13^, ^30^

Other variables influencing ECMO duration in our study included ECMO configuration, severity of illness at admission measured by SAPS II score, duration of MV, and the number of blood products. We observed that VA ECMO was correlated with shorter durations compared to VV ECMO, consistent with findings by Aubron et al.^31^ The shorter duration of VA‐ECMO can be attributed to its primary indication for acute cardiac failure, which typically involves planned intervention for cardiac recovery. In contrast, VV‐ECMO primarily supports pulmonary function, often requiring longer durations for lung healing.^32^, ^33^ Increased blood product transfusion has been correlated with prolonged ECMO duration in previous studies. Ang et al.^34^ reported positive correlations between the total number of RBC, PLT, and FFP transfused with ECMO duration. Their study included a smaller sample, 42 ECMO sessions, predominantly VA ECMO and did not adjust for other possible confounders. In our study, with more balanced ECMO configurations, we found positive associations between the number of RBCs and Cryo units and ECMO duration, while FFP exhibited a negative correlation and PLT transfusion did not significantly affect ECMO duration. Given the limited evidence on the correlation between blood product transfusion and ECMO duration, it's challenging to explain our findings of more FFP units transfused being associated with a shorter ECMO duration in our study. Further studies are encouraged to include these variables in adjusted models for better understanding the relationship with ECMO duration and other outcomes. Also, future studies are encouraged to consider excluding patients who remained on ECMO for reasons unrelated to their primary indication—such as awaiting transplant logistics—to enable a more accurate assessment of the relationship between transfusion practices and ECMO duration, especially since unnecessarily prolonged ECMO runs may increase the risk of complications. Although we did not specifically account for this subgroup in our analysis, the relative size and diversity of our cohort may help buffer the impact of this limitation. The direct correlation between increased disease severity and MV duration with ECMO duration in our study has not been explicitly explored in the literature. However, it has been indirectly assessed through major risk stratification scores within the ECMO literature.^35^

In our study, only ECMO configuration was associated with increased mortality risk, while MV duration and number of FFP transfusions were associated with less mortality risk. The increased MV duration and higher administration of FFP in ECMO patients, along with lower mortality, could potentially be explained by physiological mechanisms. The extended time on mechanical support may allow for better physiological recovery in critically ill patients. Additionally, the use of FFP might help optimise coagulation parameters and prevent bleeding complications, thereby reducing the risk of adverse outcomes and improving survival. In our study, VA ECMO patients had twice the risk of in‐hospital mortality compared to patients on VV ECMO. Comparable results have been reported in larger studies in the literature, such as the study conducted by Byun et al., which examined 856 patients, and the research by Deinzer et al., which involved 2016 patients. Friedrichson et al.^36^ investigated all ECMO patients (45 647) in Germany over a 10‐year period and reported mortality rates of 65.6% in VA ECMO and 53.9% in VV ECMO. The higher mortality associated with VA ECMO compared to VV ECMO may contribute to increased risk of complications, particularly in cases of cardiogenic shock, leading to higher mortality rates.^37^ Our study is the first, to the best of our knowledge, to investigate the impact of MV duration on mortality for VA ECMO patients, in addition to VV ECMO. While previous research has linked prolonged MV with increased mortality in VV‐ECMO, our study presents a different trend.^38^ This trend is further supported by recent evidence emerging after the COVID‐19 pandemic, demonstrating that MV duration is not necessarily associated with increased mortality in ECMO patients.^39^, ^40^ The effect of FFP on ECMO mortality presents conflicting evidence in the literature. While Kim et al.^41^ did not find FFP transfusion to increase the risk of mortality among VA‐ECMO patients in their adjusted analysis, Luo et al.^42^ reported a 1%–18% increased risk of mortality among 116 ECMO patients. Luo et al.'s^42^ smaller sample size, along with their use of FFP dosage in ml/kg/day as a predictor unit, may have influenced the results, potentially leading to skewed findings. Moreover, a small pilot randomised controlled trial in paediatric ECMO patients found no difference in mortality between patients who received scheduled FFP and those who did not.^43^ Although our study marginally associated FFP transfusion with decreased mortality, it is challenging to establish causality due to study design limitations and the conflicting evidence in the literature. Further large‐scale controlled trials investigating different thresholds for transfusing various blood components are warranted.

Strengths of the study lie in its ability to mirror the clinical reality of crossover transfusion thresholds from a high‐volume ECMO center. The ample sample size provided an opportunity to exclude patients who withdrew care, thereby mitigating potential bias that could affect assessments of ECMO duration or mortality. Additionally, the inclusion of a heterogeneous patient population, encompassing both VA and VV ECMO configurations, more accurately reflects real‐world practice. However, these strengths impacted our ability to allocate the sample size into development, validation, and testing cohorts to better evaluate the risk of bias and variation in our model. Furthermore, being retrospective presents a limitation, but strict adherence to inclusion criteria and adjustment for covariates using a stepwise method aimed to mitigate these weaknesses. Our final multivariable model explained 64.2% of the variance in ECMO duration, leaving ~36% unexplained—likely attributable to day‐to‐day changes in patient acuity and other bedside factors not captured in the retrospective dataset. Moreover, overlapping administration of multiple blood products and the lack of unit‐level documentation in the EHR hinder our ability to ascertain the precise clinical indication for each unit transfusion. As such, retrospective data extraction may not capture the nuanced decision‐making behind each transfusion, limiting our ability to determine whether blood product use simply reflects confounding by other unmeasured clinical variables or represents an independent driver of outcomes. For example, hemolysis and coagulopathy are recognised complications in ECMO patients, but the threshold at which they are considered clinically significant enough to warrant transfusion remains controversial and highly provider‐dependent. When abstracted retrospectively from the EHR, this introduces subjectivity. Given both this limitation and their low prevalence in our cohort, we chose not to include these variables in our predictive models to reduce potential bias. Additionally, our study did not capture more granular indicators of bleeding severity, such as drain output, macroscopic findings, or imaging‐confirmed bleeding. Bleeding was recorded only when explicitly documented as the reason for transfusion, limiting our ability to quantify its magnitude; however, it was not retained in our final model due to its minimal contribution to explaining ECMO duration. Finally, our model did not incorporate daily ECMO‐related complications, which may influence the ability to wean and thus prolong ECMO duration. Future prospective studies should consider capturing the timing and resolution of complications in relation to clinical readiness for decannulation, as many ECMO‐associated events improve with weaning rather than serving as a barrier to it. In addition to that, detailed transfusion‐specific documentation—as well as daily disease severity scoring, ECMO settings, and clinical indicators of readiness for decannulation—will be essential to disentangle these relationships and guide optimal practice. In conclusion, based on our study and literature findings, aiming for a Hgb level above 8 appears to be a reasonable option if clinical conditions permit, potentially resulting in reduced ECMO duration and fewer blood transfusions, which could improve mortality rates. Further prospective randomised trials investigating a transfusion threshold of 8 are necessary to validate our findings. However, considering the potential logistical challenges associated with conducting such trials, it would be beneficial for large multicenter observational studies, such as PROTECMO,^23^ to reanalyse their data. This reanalysis could involve conducting multivariable regression analyses to verify whether maintaining a haemoglobin level above 8 g/dL is indeed associated with a shorter duration on ECMO.

## 5 CONCLUSION

Targeting Hgb level above 8 g/dL in ECMO patients might be promising in decreasing ECMO duration and number of blood transfusions, which might lead to a decrease in mortality.

## AUTHOR CONTRIBUTIONS

All authors made substantial contributions to the study in accordance with the International Committee of Medical Journal Editors (ICMJE) guidelines. **Concept and Design:** Shailesh Balasubramanian, Mahmoud Alwakeel, Edward Soltesz, Kenneth McCurry, and Sudhir Krishnan. **Data Acquisition, Analysis, and Interpretation:** Divyajot Sadana, Mani Latifi, Chase Donaldson, and Brett Wakefield. **Manuscript Drafting and Critical Revision:** Shailesh Balasubramanian, Mahmoud Alwakeel, Divyajot Sadana, Mani Latifi, Chase Donaldson, Brett Wakefield, and Sudhir Krishnan. **Final Approval of the Manuscript:** All authors reviewed and approved the final version of the manuscript. **Accountability for Accuracy and Integrity:** All authors agree to be accountable for the accuracy and integrity of the work and have ensured that questions related to its accuracy or integrity are appropriately investigated and resolved.

## CONFLICT OF INTEREST STATEMENT

The authors have no competing interests.

## Supporting information


**Supplementary Table S1.** Stepwise Variable Selection Summary for Extracorporeal Membrane Oxygenation (ECMO) Duration Prediction Model.

## Data Availability

The data used in this study is not publicly available due to privacy and confidentiality concerns as governed by the Institutional Review Board (IRB #18‐1339) at the Cleveland Clinic.
