# Supplementary material for: Red blood cell transfusion practices in extracorporeal membrane oxygenation: A single‐center study
Source: Transfus Med. 2025 Jul 4;35(4):337–45. doi: 10.1111/tme.13154 (PMC12361832; doi:10.1111/tme.13154)
Supplement: Supplementary file 1 — Supplementary Table S1. Stepwise Variable Selection Summary for Extracorporeal Membrane Oxygenation (ECMO) Duration Prediction Model. [file TME-35-337-s001.docx]

# Supplementary

**Red Blood Cell Transfusion Practices in Extracorporeal Membrane Oxygenation (ECMO): A Single-Center Study**

Contents

# [Supplementary Table 1. Stepwise Variable Selection Summary for Extracorporeal Membrane Oxygenation](#_bookmark0) [(ECMO) Duration Prediction Model 2](#_bookmark0)

Supplementary Table 1. Stepwise Variable Selection Summary for Extracorporeal Membrane Oxygenation (ECMO) Duration Prediction Model

| **Step** | **Variable Entered** | **Retained?** | **Sig Prob** | **Seq SS** | **RSquare** | **Cp** | **p** | **AICc** | **BIC** |
| --- | --- | --- | --- | --- | --- | --- | --- | --- | --- |

|  | ECMO configuration (VA vs |  | | | | | | | |
| --- | --- | --- | --- | --- | --- | --- | --- | --- | --- |
| 1 | VV) | Yes | <0.0001 | 1827.94 | 0.1032 | 304.9 | 2 | 1479.58 | 1489.43 |
| 2 | Hemoglobin Group | Yes | <0.0001 | 1360.30 | 0.1801 | 263.56 | 3 | 1463.3 | 1476.4 |
| 3 | RBC units | Yes | <0.0001 | 4592.55 | 0.4394 | 119.25 | 4 | 1387.45 | 1403.76 |
| 4 | Mechanical Vent Duration | Yes | <0.0001 | 1505.48 | 0.5244 | 73.282 | 5 | 1355.85 | 1375.37 |
| 5 | FFP units | Yes | <0.0001 | 1157.09 | 0.5898 | 38.418 | 6 | 1327.7 | 1350.39 |
| 6 | Cryoprecipitate units | Yes | <0.0001 | 980.64 | 0.6452 | 9.175 | 7 | 1300.13 | 1325.98 |
| 7 | SAPS II Score | Yes | 0.0104 | 206.27 | 0.6568 | 4.603 | 8 | 1295.48 | 1324.46 |
| 8 | BMI | No | 0.0960 | 85.52 | 0.6616 | 3.878 | 9 | 1294.78 | 1326.88 |
| 9 | Platelets | No | 0.1729 | 56.94 | 0.6649 | 4.064 | 10 | 1295.06 | 1330.24 |
| 10 | Atrial Fibrillation | No | 0.2159 | 46.79 | 0.6675 | 4.574 | 11 | 1295.69 | 1333.94 |
| 11 | Age | No | 0.2070 | 48.50 | 0.6702 | 5.029 | 12 | 1296.28 | 1337.57 |
| 12 | Gender | No | 0.2804 | 35.42 | 0.6722 | 5.9 | 13 | 1297.33 | 1341.65 |
| 13 | Hgb subgroups (ns) | No | 0.4087 | 20.76 | 0.6734 | 7.239 | 14 | 1298.93 | 1346.23 |
| 14 | CAD | No | 0.5213 | 12.54 | 0.6741 | 8.839 | 15 | 1300.84 | 1351.11 |
| 15 | VTE | No | 0.5038 | 13.66 | 0.6749 | 10.404 | 16 | 1302.73 | 1355.95 |
| 16 | HTN | No | 0.6867 | 4.99 | 0.6752 | 12.245 | 17 | 1304.96 | 1361.1 |
| 17 | LVEF | No | 0.7249 | 3.82 | 0.6754 | 14.123 | 18 | 1307.25 | 1366.28 |
| 18 | Bleeding | No | >0.9 | 0.50 | 0.6754 | 15.8 | 19 | 1309.8 | 1370 |

Seq SS = sequential (Type I) sum of squares contributed by the variable at the moment it enters the model. R Square = cumulative adjusted R² after the variable is added.

Cp = Mallows’ Cp statistic; lower values closer to p indicate a better balance of fit and parsimony. p = total number of parameters in the model at that step (including the intercept).

AICc = small-sample corrected Akaike Information Criterion; BIC = Bayesian Information Criterion.

Variables were entered with forward stepwise selection. A variable is marked **Yes** when its addition lowers both AICc and BIC (steps 1–7); from

step 8 onward, each added variable increased BIC and conferred minimal ΔR², so the variable was not retained.
